# Linking Soil Properties to Mangrove Structural Parameters Across a Disturbance Gradient in the Northern Veracruz, Mexico

**DOI:** 10.1002/pei3.70176

**Published:** 2026-06-22

**Authors:** A. de J. Basánez‐Muñoz, A. Guillermo Jordán‐Garza, A. Serrano, R. Twilley

**Affiliations:** ^1^ Facultad de Ciencias Biológicas y Agropecuarias, Cuerpo Académico Manejo de Ambientes Marinos y Costeros, Universidad Veracruzana Tuxpan Mexico; ^2^ Coral Reefs Laboratory Facultad de Ciencias Biológicas y Agropecuarias, Cuerpo Académico Ecosistemas Costeros, Universidad Veracruzana Tuxpan Mexico; ^3^ Observatorio Marino y Costero Facultad de Ciencias Biológicas y Agropecuarias, Universidad Veracruzana Tuxpan Mexico; ^4^ Department of Oceanography and Coastal Sciences Luisiana State University Baton Rougem Louisiana USA

**Keywords:** disturbance, mangrove forest, mangrove structure, principal component analysis, redundancy analysis, resources, soil properties

## Abstract

Mangrove composition and structure vary with soil properties and alterations in these conditions may result in forest degradation. This study examines how soil properties relate to mangrove structure across three disturbance states. The study was conducted within the Ramsar Site 1602 in Veracruz, Mexico. Soil properties (organic matter, organic carbon, total nitrogen, salinity, pH, and conductivity) and structural parameters (density, basal area, height, and diameter at breast height) were measured in 100 m^2^ plots along four transects perpendicular to the coast. Plots represent apparently undisturbed (AU), semi‐disturbed (SD), and disturbed (D) states. Differences in soil properties and structural parameters between forest states were first assessed using principal component analysis and PERMANOVA. Then, the relationship between forest structure and soil properties was investigated using redundancy analysis (RDA). Differences in soil and forest structural characteristics were significant between all disturbance states. AU plots had higher tree density, basal area, height, and richness and complexity index than SD plots, while no living trees were found in D. The RDA showed that silt content and soil pH were the main environmental factors significantly associated with variation in mangrove structure. Distance from the lagoon edge towards the coastline had a marginal effect and the overlap of SD and D plots indicated that the spread of forest decline was associated with changes in soil properties. Effective mangrove conservation requires soil restoration measures to prevent further degradation.

## 1 Introduction

Mangrove forests are highly productive coastal ecosystems that provide essential services, including carbon sequestration, shoreline protection, biodiversity conservation, and support for coastal fisheries (Friess et al. 2020; Lovelock et al. 2025). Despite their ecological and socioeconomic importance, mangroves are increasingly threatened by land‐use change, pollution, hydrological alteration, and climate change, making their conservation a global priority (de Lacerda et al. 2019). Understanding the factors that shape mangrove composition and structure is therefore critical for assessing ecosystem condition and guiding management efforts (Feller et al. 2010). Additionally, the characterization of mangrove forests requires the study of environmental parameters between different mangrove types (Mira et al. 2019). Mangrove species zonation patterns exhibit a spatial arrangement characterized by an ordered sequence from the shoreline towards the inner forest, reflecting species‐specific adaptations to gradients of salinity, edaphic conditions, and tidal inundation (Olthoff et al. 2016; Zamora‐Tovar et al. 2025).

The main abiotic environmental factors influencing mangrove species distribution include microtopography, salinity, soil flooding, hydrological cycle, and other soil physicochemical properties such as phosphorus and nitrogen availability, pH, redox potential, and sulfide concentrations (Twilley 1998; Estrada et al. 2013; Datta and Deb 2017; Bomfim et al. 2018; Cui et al. 2025). Soil composition directly influences physicochemical conditions and results from the interaction of geological processes operating across multiple temporal scales past and present (Ferreira et al. 2010). As a regulatory mechanism, species distribution is influenced by competition for resources (nutrients) and salt tolerance (Duke et al. 1998). Low interstitial salinity (< 21 PSU) combined with high nutrient availability promotes improved structural development of mangrove forests (Camacho‐Rico et al. 2021). Understanding nutrient concentration, composition and availability within mangrove ecosystems is essential, as these parameters can vary spatially depending on local conditions. In this context, physicochemical characteristics such as pH and nutrient concentrations in soils and interstitial water provide insights into species adaptability to environmental change, as well as mangrove establishment and colonization processes (Vilarrúbia 2000; Sherman et al. 2003; Guzmán‐Sánchez et al. 2023).

Numerous studies have highlighted the importance of monitoring environmental parameters that influence mangrove richness and abundance, which in turn drive species‐specific spatial distributions (Feller et al. 2003; Rovai et al. 2021; Amores et al. 2024). Changes in water and soil composition are widely recognized as key factors affecting mangrove ecosystem functioning and survival (Wimmler et al. 2021; Dookie et al. 2023). Although the relationship between vegetation and soil properties in natural mangrove areas is well documented (Ramírez‐Fuentes and Trujillo‐Tapia 2015; Ahmed et al. 2023), studies assessing whether similar relationships exist between mangrove growth in restored systems remain limited (Salmo et al. 2013). While hydrological processes can often be restored relatively quickly through appropriate design (e.g., within one year), soil‐dependent properties and processes typically require much longer periods—ranging from decades to centuries—to resemble those of natural or intact mangrove forests (Baldwin 2017).

Human intervention has altered hydrological connectivity in many coastal environments, leading to the loss of mangrove tree cover and ecosystem degradation (Pérez‐Ceballos et al. 2020). The construction of roads, causeways, embankments, and transmission corridors, as well as wetland filling or dredging—even in areas adjacent to mangroves—can disrupt water flow, thereby altering flooding regimes and mangrove soil conditions (Jaramillo et al. 2018).

In the northern state of Veracruz a formerly continuous mangrove forest was fragmented into three sectors (southern, central, and northern) following the construction, in 1998, of three embankments supporting transmission towers for a thermoelectric complex (Basáñez‐Muñoz et al. 2021). This infrastructure caused tree mortality across more than 42 ha and led to the establishment of distinct disturbance sites. These sites were classified in situ by Vovides et al. (2011) in three disturbance categories: apparently unaffected, affected (some tree mortality), and degraded (total tree mortality). Hydrological connectivity was partially restored in 2011, when openings were created in the embankments to allow water flow into the area. We hypothesize that soil properties constitute a persistent legacy of past hydrological disruption and are linked to present day mangrove structure. Specifically, we assume that disturbance categories will differ in soil physicochemical properties and soil characteristics. Lower structural development of the forest will be associated with greater changes in soil properties rather than by the partial recovery of hydrological connectivity alone. This study aims to improve our understanding of long‐term mangrove resilience and to inform restoration strategies in altered coastal ecosystems.

## 2 Methods

### 2.1 Study Area

**FIGURE 1:**
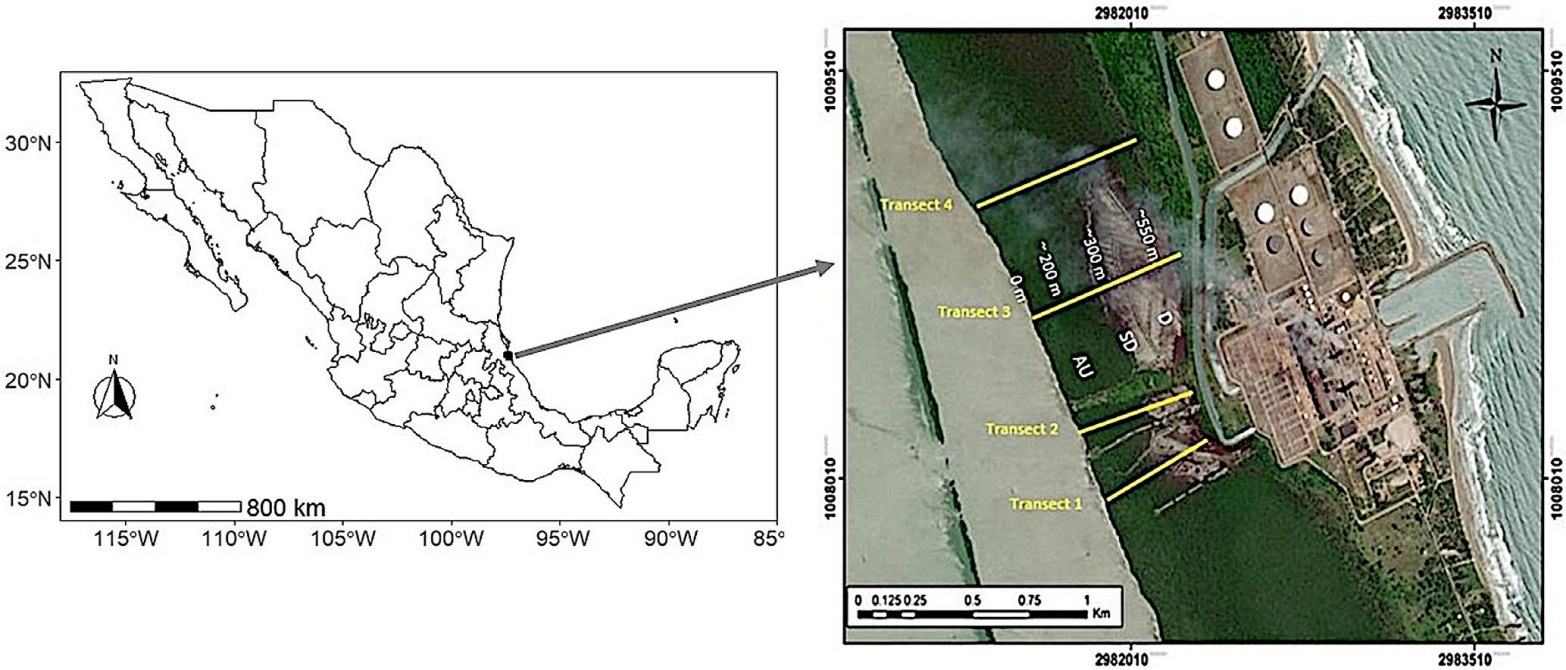
Study area in the northern state of Veracruz, Mexico. The satellite image shows the types of disturbance and distance from the lagoon to the coast: Apparently undisturbed plot (UA, 0 to 200 m), semi‐disturbed (SD, > 200 to 300 m) and Disturbed (D, > 300 m). Yellow lines show the approximate location of each transect.

**FIGURE 2:**
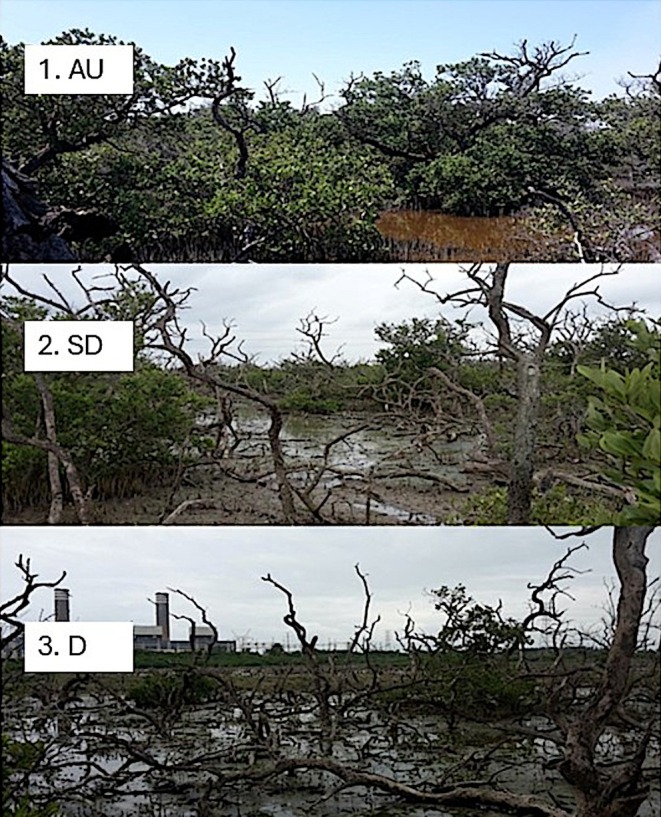
Photographs exemplifying the differences between (1) Apparently unaffected forest (AU), with all trees alive, (2) Semi disturbed forest (SD), with some death trees and shallower water, and (3) Disturbed forest (D), mostly with death trees.

The study was conducted in the Ecological Reserve of the “Presidente Adolfo López Mateos” Thermoelectric Complex (21°00′249.59″ N; 97°20′28.12″ W), located within the Ramsar Plot No. 1602 “Mangroves and Wetlands of Tuxpan” in the northern state of Veracruz, Mexico (Figure 1). In 1998 the construction of the thermoelectric facility resulted in the interruption of hydrological flow and caused tree mortality that has increased through time. Despite that hydrological connectivity was partially restored in 2011 mortality continues and in 2018, the mangrove zone that showed disturbance was approximately 42 ha (Figure 1). The dominant mangrove species in the area include *Rhizophora mangle* L., *Avicennia germinans* (L.) L., and *Laguncularia racemosa* (L.) Gaertn. By walking into the mangrove from the edge of the Tampamachoco lagoon inward to the coastline and to the end of the forest, three different types of disturbances could be visually determined by the number of trees with partial or total mortality (Figure 2). Basáñez‐Muñoz et al. (2021) showed that the forest structure significantly differed between these three levels of perturbation due to different population dynamics of the tree species on each disturbance type. The first type of disturbance named “apparently unaffected” (AU) is adjacent to the Tampamachoco lagoon and extends from the lagoon border (0 m) to approximately 200 m inward in direction to the coastline and shows no apparent tree mortality (Figure 1). Then, from 200 to 300 m inward a “semi‐disturbed” (SD) areaexhibits trees with evident signs of stress shown by leafs mortality on upper and some lower branches. Finally, the “disturbed” (D) area extends from 300 to approximately 550 m inward and is characterized by total tree mortality (Figure 2). Vovides et al. (2011) and Basáñez‐Muñoz et al. (2021) working on the same area used a similar classification, moreover similar disturbances have been reported in Cozumel and the Términos lagoon with areas apparently unaffected, a transition zone and a degraded area (López‐Adame et al. 2025).

### 2.2 Fieldwork

To encompass the entire affected area, four linear transects were systematically established from south to north along the affected area of the mangrove forest separated ~200 m from each other (Figure 1). Each transect began near the Tampamachoco lagoon border (0 m) and was laid to the northeast, towards the coastline (Figure 1). The first two transects had a length of 550 m, whereas the other two were 600 m long, reflecting variation in the forest's topographic width (Figure 1). Within each transect, 100 m^2^ quadrats were established every 50 m for a total of 12 quadrats on the first and second transects and 14 on the third and fourth transects. All individuals on the quadrats with a diameter > 2.5 cm were measured at 1.30 m above the ground for the diameter at breast height (DBH) using a metric tape, and their total height using a clinometer HAGA. For *Rhizophora mangle* trees, the diameter was measured 30 cm above the last adventitious root. To determine soil texture and estimate the percentages of organic matter (% OM), organic carbon (% OC), total nitrogen (% Nt), salinity (psu), conductivity (μS/cm), and pH, soil samples of 1 kg were collected at a depth of 30 cm in each quadrat along the transect. The samples were placed in plastic bags, labeled, and taken to the Soil Laboratory of the Faculty of Biological and Agricultural Sciences at the Universidad Veracruzana in Tuxpan, Veracruz.

### 2.3 Laboratory Work

The analyses of soil samples followed the protocols established on Mexican laws (NOM) for different soil analyses: texture analysis using the Bouyoucos method (AS‐09 in SEMARNAT 2002), organic carbon content (% C.O.), and organic matter content (% M.O.) were determined using the Walkley and Black method (AS‐07 in SEMARNAT 2002), and total nitrogen content (% Nt) was estimated considering 5% organic matter (Plaster 2000). Electrical conductivity was measured in the saturation extract (AS‐18 in SEMARNAT 2002) using a conductivity meter. The pH and temperature (AS‐24 in SEMARNAT 2002) were registered with a multiparameter (Tester TM 35 series), and salinity was obtained through an indirect method using a conductivity meter.

### 2.4 Statistical Analysis

To examine multivariate patterns in soil physicochemical properties across the three disturbance categories: (1) apparently unaffected (AU), (2) semi‐disturbed (SD), and (3) disturbed (D), soil environmental variables (including soil composition (sand, clay, or silt), OM, OC, TN, salinity, pH, conductivity, and the distance of the plot from the lagoon border towards the coastline) were first inspected and standardized to account for differences in measurement units. A principal component analysis (PCA) based on a correlation matrix was used to examine patterns of variation in soil properties. Differences in soil properties among disturbance levels were tested using PERMANOVA using Euclidian distances and 999 permutations (Anderson 2001).

Because no living trees were recorded in disturbed sites, comparisons of the forest structural attributes (tree density, basal area, height, richness, DBH, and complexity index) were conducted between the AU and SD categories using the same PCA and PERMANOVA approach.

To identify the variables most strongly associated with the multivariate patterns, vectors of the original variables were fitted onto the first two PCA axes using the envfit function with 999 permutations. This procedure allowed the identification of variables significantly correlated with the major gradients represented by the ordination axes. PCA was selected because it is an appropriate linear‐ordination method for continuous environmental and structural variables widely used to reduce dimensionality and identify dominant gradients in ecological datasets with approximately linear relationships among variables (Legendre and Legendre 2012).

Finally, a redundancy analysis (RDA) was used to explore the relationships between mangrove forest structural attributes and soil environmental variables. RDA is a constrained ordination technique that directly relates multiple response variables to a set of explanatory variables, allowing the identification of environmental gradients associated with variation in ecological attributes (Makarenkov and Legendre 2002). Prior to the analysis, results from the PCA and vector fitting were used to guide variable selection. Variables that were not statistically significant or that exhibited strong collinearity with other variables were excluded. This approach has been widely applied in mangrove ecology to identify environmental drivers of vegetation structure and ecosystem variability across disturbance gradients (Alongi 2009; Toosi et al. 2022).

All multivariate analyses were performed in R using the vegan package (Oksanen et al. 2025).

## 3 Results

**FIGURE 3:**
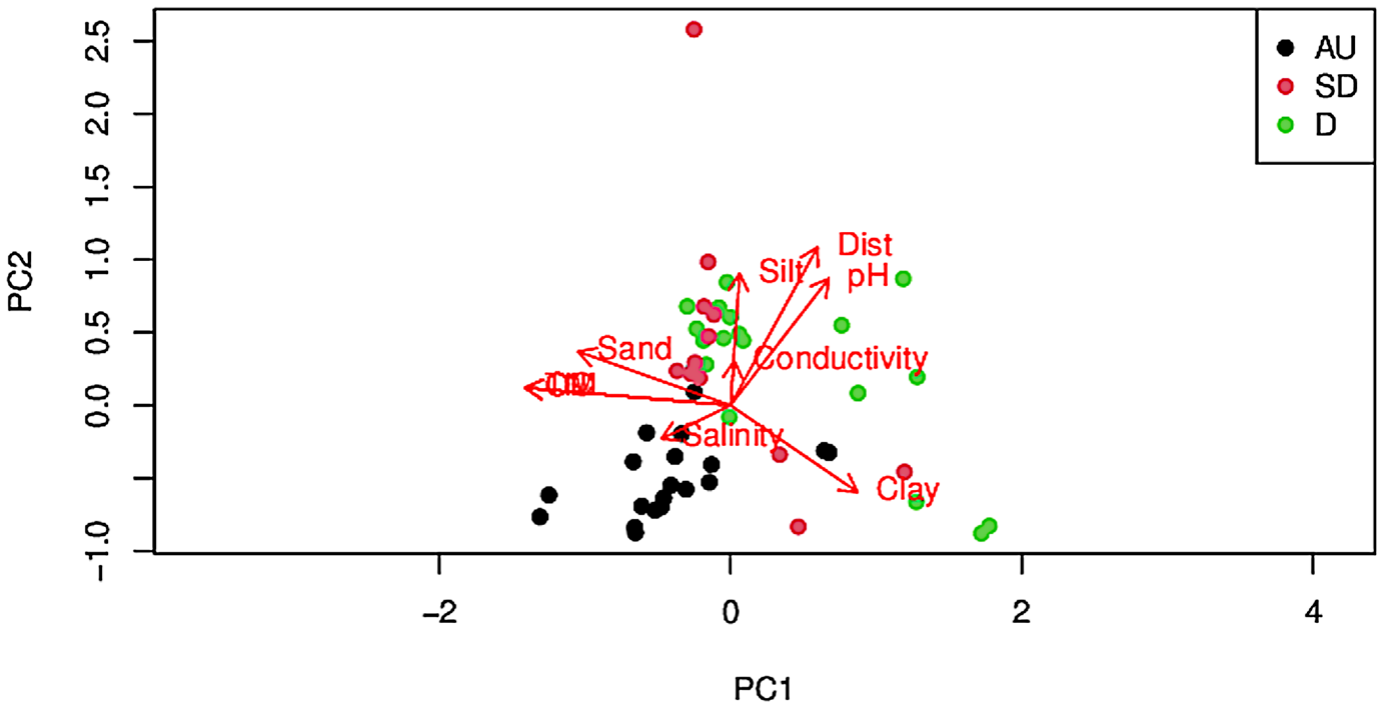
Principal component analysis (PCA) ordination showing variation in soil properties across mangrove plots under three disturbance categories. AU (apparently undisturbed, black), SD (semi‐disturbed, red) and D (disturbed, green). The first axis of the ordination explained 40% and the second 15.5% of the variance in the dataset. Points represent sampling quadrats, and arrows indicate the direction and strength of the contribution of soil characteristics to the ordination. Variables are: Soil composition (sand, silt and clay); distance of the sample from the lagoon edge (Dist); salinity, conductivity, pH and content of organic matter (OM), total nitrogen (TN) and organic carbon (OC). The distribution of points separates between disturbance categories with some overlap, indicating a gradient in variation of soil properties with disturbance.

In the present study, a total of *n* = 576 individuals were recorded, of which 239 were *Rhizophora mangle* , 231 were *Avicennia germinans* and six were *Laguncularia racemosa* . The species *R. mangle* (*n* = 239), *A. germinans* (*n* = 231) and *L. racemosa* (*n* = 6) were found at the apparently undisturbed plots (AU), while *A. germinans* (*n* = 100) was dominant at the semi‐disturbed plots (SD). No living trees were found at the disturbed plots (D). The principal component ordination of soil properties across the three disturbance categories showed a clear separation of samples pertaining to the AU, SD, and D categories (Figure 3). The first axis of the ordination explained 40% and the second 15.5% of the variance in the dataset. AU plots were primarily distributed along the negative portion of the first axis whereas D plots tended to occur towards positive values of the first axis and SD occupied an intermediate position partially overlapping with AU and D (Figure 3). A PERMANOVA showed significant differences in soil properties among disturbance levels (pseudo‐*F* = 7.39, df = 2, *p* < 0.001). No significant differences in multivariate dispersions were found (ANOVA *F* = 0.87, df = 2, *p* = 0.42). Multiple comparisons showed significant differences between AU and D plots (*p* < 0.001), AU and SD (*p* < 0.001) and SD and D plots (*p* = 0.024). The environmental vector fitting indicated that the main gradients structuring soil variability were strongly associated with organic matter and nutrient content (Table 1). Organic matter (OM), organic carbon (OC) and total nitrogen (TN) redundantly showed the strongest correlations with the ordination all aligned with the negative direction of the first axis (Table 1). Sediment texture also contributed significantly to the multivariate pattern, with sand negatively associated to the first axis, clay positively associated to the first axis and silt positively correlated to the second axis (Table 1). Distance and pH were significantly related to the ordination with AU plots closer to the lagoon edge and with lower pH values (Figure 3, Table 1). Conductivity was not significant (Table 1). Organic matter, organic carbon and total nitrogen showed the same negative loading on the first PCA axis, indicating strong collinearity among these variables (Table 1). To avoid minimizing multicollinearity only organic matter was used in the RDA.

**TABLE 1:** Contribution and significance to the ordination axes of the main gradients structuring soil variability in the principal component analysis.

Variables	PC1	PC2	*r* ^2^	*p*
Sand (%)	−0.94	0.33	0.55	0.001
Clay (%)	0.83	−0.56	0.50	0.001
Silt (%)	0.07	1.00	0.37	0.001
Salinity	−0.90	−0.44	0.13	0.04
pH	0.61	0.79	0.55	0.001
Conductivity	0.09	1.00	0.04	0.342
Organic matter (%)	−1.00	0.09	0.90	0.001
Organic carbon (%)	−1.00	0.08	0.90	0.001
Total nitrogen (%)	−1.00	0.08	0.90	0.001
Distance	0.48	0.88	0.69	0.001

**FIGURE 4:**
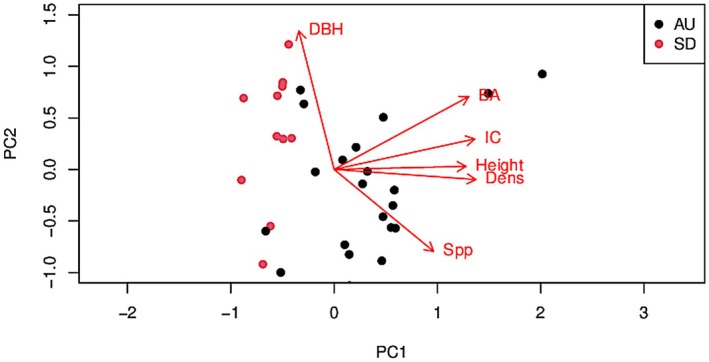
Principal Component Analysis (PCA) biplot of mangrove structural parameters comparing apparently undisturbed (AU, black) and semi‐disturbed (SD, red) plots. The first axis of the ordination explained 59.4% and the second 22.2% of the variance in the dataset. Variables are basal area (BA), index of complexity (IC), tree height, tree density (Dens), species richness (Spp) and diameter at breast height (DBH). The points distributions separate between disturbance categories with some overlap, indicating a gradient in variation of forest structure with disturbance.

For structural variables the principal component ordination showed separation between the AU and SD plots along the first axis that explained 59.4% of the variance (Figure 4). The second axis explained an additional 22.2% of the variance. AU plots were distributed to the right of the ordination and SD to the left with little overlap. Variables such as basal area, tree height, IC index, richness, and density increased towards AU plots. DBH was more strongly related to the second axis. A PERMANOVA showed significant differences in structural variables among disturbance levels (pseudo‐*F* = 12.4, df = 1, *p* < 0.001). No significant differences in multivariate dispersions were found (ANOVA *F* = 3.2, df = 1, *p* = 0.08). All structural variables were significantly associated to the ordination space (Table 2). Variables such as basal area, tree height, IC index, richness, and density were positively aligned with the first axis that represents a gradient of increasing structural developments.

**TABLE 2:** Contribution and significance to the ordination axes of the main gradients of forest structure in the principal component analysis.

Variables	PC1	PC2	*r* ^2^	*p*
Density	0.99745	−0.07135	0.8327	0.001
Basal area	0.87919	0.47647	0.965	0.001
Height	0.99973	0.02317	0.7168	0.001
Richness	0.77127	−0.63651	0.6861	0.001
Diameter at breast height	−0.24651	0.96914	0.8445	0.001
Index of complexity	0.97722	0.21221	0.8533	0.001

**FIGURE 5:**
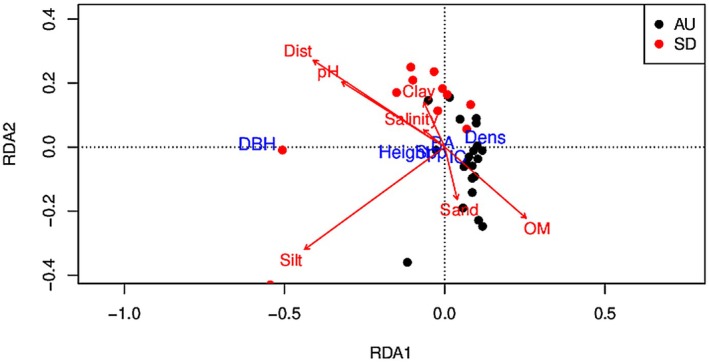
Redundancy analysis (RDA) illustrating the relationships between mangrove structural parameters (blue) and soil properties (red) across apparently undisturbed (AU, black) and semi‐disturbed (SD, red) plots. The first canonical axis explained 69.34% and the second axis 0.56% of total variation. Soil variables are: Soil composition (sand, clay, and silt), pH, organic matter (OM), salinity and distance (Dist) of the sample to the lagoon edge. Structural variables are basal area (BA), index of complexity (IC), tree height, tree density (Dens), species richness (Spp), and diameter at breast height (DBH). The distribution of samples indicates partial separation between disturbance conditions, reflecting a gradient in soil structure relationships associated with disturbance.

A RDA was performed using significant and uncorrelated soil variables from the PCA (Figure 3, Table 1; texture, salinity, pH, organic matter, and distance from the lagoon edge) and structural variables (tree density, basal area, height, richness, DBH and complexity index). The constrained model explained 70.1% of the inertia in forest structure, and the RDA was statistically significant (*F* = 8.05, *p* = 0.001, 999 permutations). The first canonical axis explained 98.8% of the constrained inertia and 69.3% of the total variation and was significant (*F* = 58.05, *p* = 0.002); the remaining axes were not significant. In the RDA ordination, AU plots were located at the positive values of the first axis and at positive and negative values of the second axis. In contrast, SD plots were located mainly at the negative values of the first axis and positive values of the second axis but with clear overlap with some AU plots (Figure 5). The first axis was positively associated with gradients in organic matter and sand and negatively with pH, silt, clay, salinity and distance. Structural variables clustered near the center. Sequential permutation tests of individual environmental variables indicated that silt content (*F* = 38.03, *p* = 0.001) and soil pH (*F* = 12.48, *p* = 0.001) were the main environmental factors significantly associated with variation in mangrove structure. Distance showed a marginal effect (*F* = 3.35, *p* = 0.065) and was correlated to pH; other variables did not contribute significantly to the model (Table 3). Vector fitting of structural variables onto the RDA showed that all forest attributes were significantly associated with the environmental gradients (Table 4).

**TABLE 3:** Results of a permutational analysis of variance for constrained ordination on the redundancy analysis showing the significance of individual environmental variables.

Variables	df	Variance	*F*	*p*
Sand	1	0.000036	0.3035	0.577
Clay	1	0.000075	0.6356	0.435
Silt	1	0.004513	38.0306	0.001
Salinity	1	0.000007	0.066	0.871
pH	1	0.001481	12.4803	0.001
OM	1	0.000177	1.4929	0.232
Distance	1	0.000398	3.3589	0.065
Residual	24	0.002848		

**TABLE 4:** Contribution and significance to the ordination axes of the forest structural variables in the redundancy analysis.

Variables	RDA1	RDA2	*r* ^2^	*p*
Density	0.98	0.17	0.94	0.001
Basal area	−0.74	0.66	0.37	0.003
Height	−0.97	−0.21	0.91	0.001
Richness	−0.84	−0.53	0.76	0.002
Diameter at breast height	−0.99	0.017	0.99	0.001
Index of complexity	0.69	−0.71	0.50	0.003

## 4 Discussion

Significant differences in soil properties and structural parameters were found between the three characterized disturbances. These differences can be attributed to modifications related to hydrological flow caused by the construction of power‐generating facility embankments (Vovides et al. 2011; Basáñez‐Muñoz et al. 2021). In the principal component ordination based on soil properties (Figure 3), the apparently undisturbed plots (AU) separated from the semi‐disturbed (SD) and disturbed (D) plots. Although some overlap was evident (Figure 3), a PERMANOVA analysis showed that the differences between all three levels were significant. Soil properties in mangrove forests are sensitive to disturbance and land‐use changes; degraded systems have shown reduced organic matter, altered nutrient dynamics, and shifts in sediment composition (Alongi 2009; Santos‐Andrade et al. 2021). Similarly, the forest in the AU plots exhibited better indicators of density, basal area, height, and complexity index than the SD plots. It is highly likely that alterations or modifications in hydrology have pushed these forests beyond their ecological tolerance range, leading to degradation and total loss of individuals at the D plots (Zaldívar‐Jiménez et al. 2010; Pérez‐Ceballos et al. 2017). Other studies have reported significant differences between disturbed and undisturbed forest plots, with the undisturbed plots showing better development, for example, Kairo et al. (2002) in Mida and Gazi, Kenya, and Pambala and Galle in Sri Lanka, and Kihia (2014) in Gazi Bay, Kenya, as well as Nwobi and Williams (2021) in the Niger Delta, Nigeria. Piponiot et al. (2022) showed how tree size is related to the environmental conditions of the site and helps understand the response of the system to external forcing factors.

The apparently undisturbed plots (AU) were composed of *Rhizophora mangle* , *Avicennia germinans* , and *Laguncularia racemosa* . However, in the semi‐disturbed plots (SD), only *Avicennia germinans* was present. In the AU plots, higher values of salinity, nitrogen, carbon and organic matter and lower pH were observed (Figure 3), indicating the typical characteristics of a fringe mangrove (Agudelo et al. 2015). Conversely, opposite values were observed in the SD plots, indicating a basin‐type forest. Cintrón‐Molero and Schaeffer‐Novelli (1992) reported that the genus *Avicennia* is more tolerant to stressful environmental factors and, therefore, can be found abundantly in areas with disturbances related to human activities. The individuals found in the semi‐disturbed plot (SD) had an average DBH of 10.14 cm, but their average height was 2.83 m due to the stress factor of salinity, which caused the death of upper canopy branches and their subsequent detachment from the tree. Vovides et al. (2011) reported an average DBH of 10.6 cm and a height of 3.5 m for trees in a severely degraded mangrove area of their study. The decomposition rates of *R. mangle* and *L. racemosa* leaves are slower than those of *A. germinans* leaves (Agudelo et al. 2015; Lima and Colpo 2014), resulting in carbon storage in the soil (Valdés‐Velarde et al. 2011). This increases the percentage of organic carbon (OC %) in basin‐dominated forests by *A. germinans* as they retain more organic matter (Prasad and Ramanathan 2008). Another important parameter in the study area was the pH, which was lower in AU (pH 5.42) and higher in D (pH 6.43). Almahasheer et al. (2016) reported that nutrient availability in mangroves is strongly regulated by the soil pH. Similarly, Cooray et al. (2021) in Sri Lanka observed that the concentration of micronutrients decreased as pH increased. Extended periods of inundation can also affect the soil pH and redox potential, especially in organic‐rich soils (Donato et al. 2011). Based on personal observations (A. de J. Basañez‐Muñoz), during the rainy season, the disturbed plots (D) remained flooded for several weeks, and with an organic matter percentage of 21.72 (% OM), they could be considered rich in organic matter. Overall, it is expected that soil salinity in mangroves will decrease as they move away from the water body. However, Vovides et al. (2011) reported the opposite trend in their study area, with a gradual increase in interstitial salinity from their sampling plot near the lagoon to the dead mangrove plot (increasing from ±50 to 100 psu). In the present study, salinity measurements ranged from 30.06 psu in AU to 21.72 psu in D, which can be attributed to the opening of the embankments that retained internal flow from the Tuxpan River into the mangrove. López‐Adame et al. (2025) reported a similar event in Laguna de Montecristo, Cozumel, where hydrological connectivity was disrupted, leading to water stagnation, increased salinity and physiologically stressful conditions that resulted in tree mortality. Salinity differences between AU and D can also be related to their relationship with higher accumulation of % OM (30.06 psu and 11.00% OM in AU and 21.72 psu and 8.60% OM in D) (Hapsari et al. 2020). Gutiérrez et al. (2016) studied plots with low and high disturbance levels in the Agua Brava lagoon mangroves in Mexico and found a trend of increasing organic matter in the soil of the low‐disturbance plots, along with a high concentration of organic carbon and nitrogen. This was attributed to the living trees contributing to the accumulation of organic matter, especially in areas with extensive vegetation and productivity (fallen leaves, branches, flowers, and miscellaneous). In contrast, Vovides et al. (2011) did not report significant differences in % OM, % OC, and % Nt between their undisturbed and completely degraded plots, which could be attributed to the presence of a large amount of necrotic mass in the plot, starting the decomposition process. However, variations between disturbance plots (AU and D) have important consequences for soil carbon, nitrogen and phosphorus levels (Alongi 2021; Zhu et al. 2022). Higher tree density in AU plots promotes greater organic matter accumulation, resulting in increased soil carbon concentrations (Sugiana et al. 2024). In contrast, soil disturbance associated with tree loss in D plots, along with prolonged flooding conditions, can negatively affect nitrogen and phosphorus cycling (Chen et al. 2021). The concentration of these resources (% OM, % OC, and % Nt) depends on the residence time of organic matter in the soil and the type of vegetative residue (Gutiérrez et al. 2016; Grueters et al. 2021). Organic carbon is responsible of the formation of plant biomass, supports the physical structure of plants, and serves as carbon reservoir to mitigate climate change (Sugiana et al. 2024).

Similar to the findings of Vovides et al. (2011), high pH continues to be a stress factor in the semi‐disturbed (SD) and disturbed (D) plots in the study area. The authors reported a pH of 6.8, in their severely degraded plot in 2011, and in 2018, it remains at 6.47 in the semi‐disturbed plots (SD). The regulation of various biochemical processes and the influence on nutrient availability are attributed to soil pH (Oshunsanya et al. 2019). Salinity, which was reported to be 100 psu in 2011 before the opening of embankments, decreased to 22 psu in 2018. The % Nt in 2011 was 1.2, which decreased to 0.432018. PCA showed a clear trend of the soil properties with the distance of the plots from the edge of the lagoon. Considering that the disturbed plots (D) start to manifest at approximately 300 m from the lagoon edge, it can be inferred that the mangrove degradation process has gradually occurred towards the mangrove near the lagoon. This process was verified by observing satellite images available on Google Earth from 2004 to 2018.

The RDA revealed a correlation between mangrove forest structural attributes and gradients in soil properties. The first canonical axis accounted for most of the explained variation and represented the primary gradient associated with differences in soil properties and mangrove structural attributes, separating plots along silt content and pH. These associations are consistent with patterns reported in mangrove ecosystems, where hydrological conditions and salinity are often linked to both soil characteristics and vegetation structure (Alongi 2009; Kristensen et al. 2008; Elmahdy and Ali 2022). However, because hydrological variables were not measured in this study, their influence cannot be evaluated explicitly. The RDA ordination does not allow inference of causal relationships between soil properties and mangrove structure; instead, the analysis indicates that variation in mangrove structural attributes is associated with variation in soil properties across the disturbance zones. The direction and underlying mechanisms of these relationships remain unresolved.

It is essential to establish permanent monitoring sites to determine if tree degradation continues as a chronic event in the study area despite management efforts. It is noteworthy that the disturbance initiated by the establishment of the power plant triggered a cascade of changes that have resulted in the disturbance regimes characterized in this study, with tree mortality progressively increasing over time. Recent management efforts have attempted to restore hydrological connectivity in the area; however, it is important to emphasize that, beyond the process described here, mangroves in the region are also subjected to ongoing coastal development, pollution, deforestation, and the impacts of climate change. These multiple stressors are widely recognized as primary drivers of mangrove degradation that alter hydrology, sediment dynamics, and biogeochemical processes, ultimately compromising ecosystem structure and resilience (Alongi 2015; Akram et al. 2023).

## 5 Conclusion

The construction of the causeways in 1988 altered water circulation leading to changes in soil properties and mangrove degradation across the study area. In 2011, efforts were made to partially restore hydrological circulation; yet, 7 years later we showed that changes in soil characteristics were associated with differences in mangrove forest structure among the disturbance zones. While this time frame is likely insufficient to evaluate long‐term trajectories in a forest ecosystem, where structural changes can take decades, it is adequate to detect the ecological consequences of altered hydrology and the persistence of disturbance effects on both soils and vegetation. The observed patterns in this study reflect both the immediate mortality caused by hydrological alteration, the gradual but continuous deterioration of soil characteristics, and the subsequent performance of trees under altered environmental conditions. It is crucial to continue monitoring the area, especially near the lagoon and to the north of the study area where the mangrove is now showing signs of deterioration. Restoring soil properties, in addition to other restoration and management policies, could contribute to the conservation of mangroves and their complex ecosystem for the future.

## Funding

The authors have nothing to report.

## Conflicts of Interest

The authors declare no conflicts of interest.

## Data Availability

The data that support the findings of this study are openly available in GitHub at https://github.com/ajordangarza/Linking‐Mangrove‐Soil‐and‐Structural‐Parameters.git.
